# Preliminary analysis to estimate the spatial distribution of benefits of P load reduction: Identifying the spatial influence of phosphorus loading from the Maumee River (USA) in western Lake Erie

**DOI:** 10.1002/ece3.6160

**Published:** 2020-04-12

**Authors:** James H. Larson, Enrika Hlavacek, Nathan DeJager, Mary Anne Evans, Timothy Wynne

**Affiliations:** ^1^ Upper Midwest Environmental Sciences Center U.S. Geological Survey La Crosse WI USA; ^2^ Great Lakes Science Center U.S. Geological Survey Ann Arbor MI USA; ^3^ Center for Coastal Monitoring and Assessment National Centers for Coastal Ocean Science National Ocean Service NOAA Silver Spring MD USA

**Keywords:** cyanobacteria, HABS, Lake Erie

## Abstract

Since the early 2000s, Lake Erie has been experiencing annual cyanobacterial blooms that often cover large portions of the western basin and even reach into the central basin. These blooms have affected several ecosystem services provided by Lake Erie to surrounding communities (notably drinking water quality). Several modeling efforts have identified the springtime total bioavailable phosphorus (TBP) load as a major driver of maximum cyanobacterial biomass in western Lake Erie, and on this basis, international water management bodies have set a phosphorus (P) reduction goal. This P reduction goal is intended to reduce maximum cyanobacterial biomass, but there has been very limited effort to identify the specific locations within the western basin of Lake Erie that will likely experience the most benefits. Here, we used pixel‐specific linear regression to identify where annual variation in spring TBP loads is most strongly associated with cyanobacterial abundance, as inferred from satellite imagery. Using this approach, we find that annual TBP loads are most strongly associated with cyanobacterial abundance in the central and southern areas of the western basin. At the location of the Toledo water intake, the association between TBP load and cyanobacterial abundance is moderate, and in Maumee Bay (near Toledo, Ohio), the association between TBP and cyanobacterial abundance is no better than a null model. Both of these locations are important for the delivery of specific ecosystem services, but this analysis indicates that P load reductions would not be expected to substantially improve maximum annual cyanobacterial abundance in these locations. These results are preliminary in the sense that only a limited set of models were tested in this analysis, but these results illustrate the importance of identifying whether the spatial distribution of management benefits (in this case P load reduction) matches the spatial distribution of management goals (reducing the effects of cyanobacteria on important ecosystem services).

## Graphical abstract

Peak cyanobacterial abundance is strongly associated with total bioavailable phosphorus (TBP) load in Lake Erie (U.S., Canada), but only in certain regions of the lake. Weak associations between cyanobacterial abundance and TBP are present in several areas of Lake Erie that are most important for the provisioning of ecosystem services (e.g., near the city of Toledo). Existing data provides little evidence that reductions of TBP will lead to reductions of peak cyanobacterial biomass in these important areas.

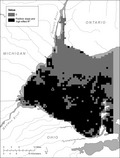

## 1 INTRODUCTION

Lake Erie is a large lake on the border between the United States and Canada. This lake, its tributaries, and river mouths feature prominently in the history of environmental regulations as an indicator of both failure and success in terms of water quality (Adler, 2002; Reitze, 1968). The western basin of Lake Erie was plagued by recurring harmful “algal” blooms (HABs) during much of the 20th century, but these HABs were reduced or eliminated by the end of the century (Allinger & Reavie, 2013). However, over the past 15 or so years, HABs have returned, mostly in the form of cyanobacterial species (especially *Microcystis* spp.; Kane, Conroy, Peter Richards, Baker, & Culver, 2014). The severity of these 21st century blooms appears to be associated with the spring loads of phosphorus (P) from the Maumee River (Bertani et al., 2016; Obenour, Gronewold, Stow, & Scavia, 2014; Wynne & Stumpf, 2015), although other explanations have also been suggested (Newell et al., 2019).

Lake Erie also provides many ecosystem services to the surrounding human communities (Allan et al., 2017). Ecosystem services are the benefits that humans obtain from ecosystems (MEA, 2003), and examples include drinking water, commercial fishing, water purification, and many related to tourism or recreation (see examples for Lake Erie in Allan et al., 2017). Many of these benefits are concentrated in nearshore areas and the areas surrounding tributary inputs (Braden, Taylor, et al., 2008; Braden, Won, et al., 2008; Elliott & Whitfield, 2011; Larson et al., 2013). Some of these services appear to be directly threatened by the continued presence of cyanobacterial blooms, including water use, commercial fishing, and recreational fishing (Bullerjahn et al., 2016; Larson et al., 2018). Due to the threats to these and other ecosystem services, an international team was assembled to identify a strategy to reduce four “eutrophication response indicators,” which included the maximum cyanobacterial biomass in the western basin of Lake Erie (Annex 4 Task Team, 2015).

The western basin of Lake Erie, where most HABs occur in Lake Erie, is dominated by inputs from two water sources: the Maumee River and the Detroit River. The Detroit River drains the upper Great Lakes (Superior, Michigan and Huron), has a high median discharge (5,200 m^3^/s), and contributes about 87% of the total water inputs to Lake Erie, but has low concentrations of P and other nutrients (Maccoux, Dove, Backus, & Dolan, 2016; Quinn & Guerra, 1986). By contrast, the Maumee River drains an intensively developed watershed, has a much lower median discharge (174 m^3^/s), and has high concentrations of P, sediment, and other nutrients (Maccoux et al., 2016; Robertson, Hubbard, Lorenz, & Sullivan, 2018). The Annex 4 Task Team (2015) focused on P reductions as a strategy that would reduce the maximum cyanobacterial biomass, using an ensemble modeling approach to identify that a 40% reduction in Maumee River total bioavailable phosphorus load (TBP, dissolved reactive P plus a portion of particulate P, Baker et al., 2014; Bertani et al., 2016) would be sufficient to reach management objectives.

While spring/early summer loads of TBP appear to have a relatively strong association with the total biomass of cyanobacteria in western Lake Erie (Bertani et al., 2016), the actual waters of the Maumee River are not mixed randomly throughout the western basin or Lake Erie as a whole. This creates a uneven spatial distribution in the distribution of primary production and thus an uneven distribution of potential effects on ecosystem services (Fang et al., 2019; Manning et al., 2019). A recent analysis by Manning et al. (2019) used regression analysis to relate the overall bloom to subregions within the western basin and demonstrated that large bloom events are not affecting all regions of the western basin equally. The previously untested implication is that the benefits from TBP load reductions will similarly not be evenly distributed across the western basin of Lake Erie. Here, we used pixel‐level regression models to identify where benefits of the target TBP load reductions will be greatest and least.

## 2 METHODS

### 2.1 Cyanobacterial abundance

A satellite‐derived cyanobacterial index (CI; cells/ml) was used as an indicator of cyanobacterial abundance. This CI is described in Wynne, Stumpf, Tomlinson, and Dyble (2010) and Stumpf, Wynne, Baker, and Fahnenstiel (2012) and is adapted to use the Moderate Resolution Imaging Spectroradiometer (Wynne, Stumpf, & Briggs, 2013). At the time of analysis, the CI was available for the years 2002–2016. To compensate for shallow optical depth, satellite images are taken during low wind conditions when it is assumed the dominant *Microcystis* will float to the surface (Wynne et al., 2010). A single CI value was calculated for each pixel over each 10‐day period starting in June and ending in October. Individual pixels are 1,100 × 1,100 m. Edge pixels were ignored for this analysis. Limitations of the CI are discussed in the above‐referenced studies, but include data loss due to cloud cover and the potential for community composition shifts to magnify or obscure important variation in cell density (Binding, Zastepa, & Zeng, 2019). We used the maximum CI index value at each pixel, as this was the most analogous pixel‐level response to the peak bloom size measurement used in other modeling efforts (e.g., Bertani et al., 2016). For years where the CI was never above the detection limit, we set the maximum equal to zero. Pixels masked by cloud cover were ignored. Maximum CI index at a given pixel is also statistically easy to estimate compared with mean or variance because of the large number of values below the detection limit that occur at most pixels and because of missing data due to cloud cover.

### 2.2 Phosphorus loads from the Maumee River

Nutrient loads were calculated from data collected by the National Center for Water Quality Research’s (NCWQR) tributary monitoring program (methods and data available online at https://ncwqr.org/). Total bioavailable phosphorus is often used to predict the extent or severity of cyanobacterial blooms in Lake Erie (Bertani et al., 2016; NOAA, 2019). Total bioavailable phosphorus combines soluble reactive phosphorus (SRP) and particulate P, but weights particulate P as much less available to support phytoplankton than SRP (Baker et al., 2014; Bertani et al., 2016). Several studies have found that spring TBP loads (March 1–July 31) are strongly correlated with peak bloom size (Annex 4 Task Team, 2015; Bertani et al., 2016; Obenour et al., 2014), so we used published estimates of the spring TBP load (available online at http://data.glos.us/maumee/). A recent study found that reduced forms of nitrogen are strongly associated with cyanobacteria in this ecosystem as well (Newell et al., 2019). We used the NCWQR data to calculate March–July nitrate + nitrite load (NO23; oxidized N), total Kjeldahl nitrogen (TKN; reduced N), and the ratio of TKN:NO23 (which are the predictors used in Newell et al., 2019).

### 2.3 Air temperature

Air temperature was derived from NOAA’s website (https://www.ncdc.noaa.gov/cdo-web/), weather station # USW00004848 (Toledo Metcalf Field, Ohio, U.S.). Monthly averages from March to September were multiplied by the number of days for that month and then divided by the total number of days from March to September to get a weighted mean temperature.

### 2.4 Discharge

Discharge for the Maumee River was downloaded from the U.S. Geological Survey’s (USGS) monitoring gage at Waterville, Ohio (USGS 04,193,500; https://doi.org/10.5066/F7P55KJN). Daily average discharge was summed for the entire period from March 1 to July 31 and divided by the number of days to get a season‐long average daily discharge value.

### 2.5 Regression analysis

For each pixel, a simple linear regression was performed between the TBP and the annual maximum CI for that pixel using the CurveFit extension to ESRI’s ArcMap software (De Jager & Fox, 2013). We focused on the regression coefficient of determination (*R* ^2^) and the slope (referred to as “*a*” in the CurveFit software) as indicators of the fit and effect size of the model relating TBP and CI. Following Cohen (1992), areas were delineated where models had an *R* ^2^ consistent with a low effect (*R* ^2^ < .09), a medium effect (*R* ^2^ = .09–.25), or a high effect (*R* ^2^ > .25). We also estimated the areas with a strong association with TBP, which was defined as having both a high positive slope (>1 *SD* from zero) and a high effect *R* ^2^ value (>.25).

**FIGURE 1:**
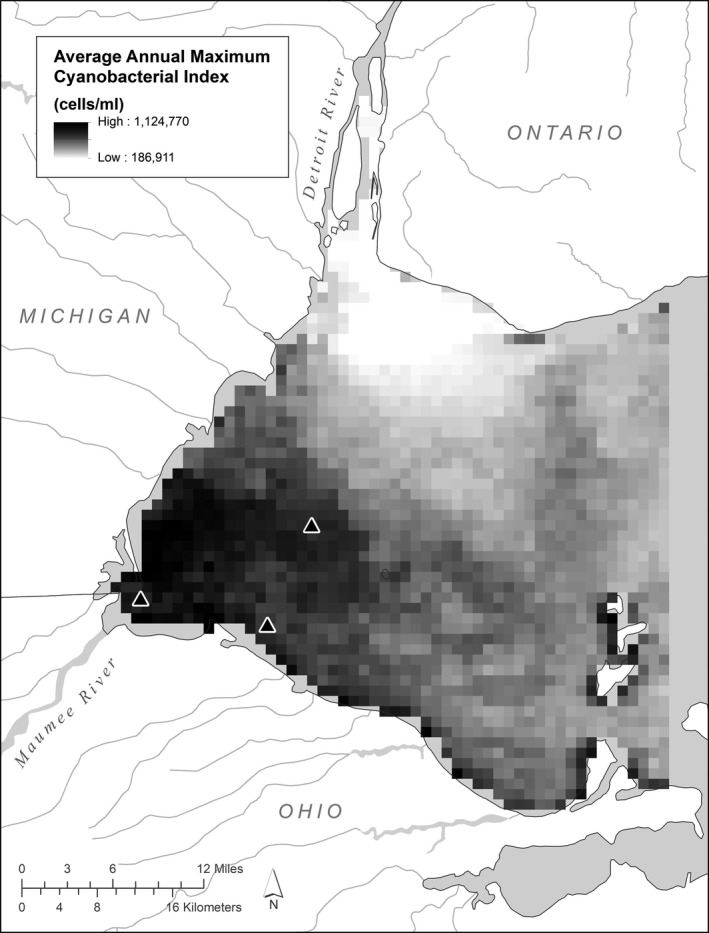
Average annual maximum cyanobacterial index values for the western basin of Lake Erie from 2002 to 2016. Triangles indicate the Maumee Bay and western basin sites that are highlighted in Figure 4

In addition, three sites represented by individual pixels were selected for more detailed analysis: (1) a site in Maumee Bay (41.7275 N, 83.4234 W), (2) a site in the open waters of the western basin (41.79737 N, 83.2044 W), and (3) a site near the Toledo water intake (coordinates not provided per USGS policy, approximate locations shown in Figure 1). Sites 1 and 2 were selected based on the results of the above regression analysis and correspond, respectively, to (a) a site where TBP loads were weakly associated with cyanobacterial abundance and (b) a site where TBP loads were strongly associated with cyanobacterial abundance. Site 3 was selected due to its particular importance for ecological services provided by Lake Erie (e.g., fresh water; Allan et al., 2017; MEA, 2003). Sites 1 and 2 are also associated with ecosystem service provision including shoreline aesthetics (site 1), recreational services (sites 1 and 2), recreational fishing (sites 1 and 2), and provisioning services (sites 1 and 2). At these sites, seven simple linear regression models were compared using Akaike's information criterion corrected for small sample size (AIC_C_; Burnham & Anderson, 1998). These models were (a) no predictor variables (null model), (b) the TBP load (repeating the pixel‐level regression from above), (c) the March–July discharge, (d) the March–September temperature, (e) the TKN load, (f) the NO23 load, and (g) the TKN:NO23 ratio. Models were fit, and AIC_C_ values were calculated using R (R Development Core Team, 2014). For the purpose of comparing effect sizes between models, standardized slopes were also estimated by scaling the data prior to model fitting using the scale() function in R. Pearson's correlation coefficients were also calculated in *R*.

## 3 RESULTS

**FIGURE 2:**
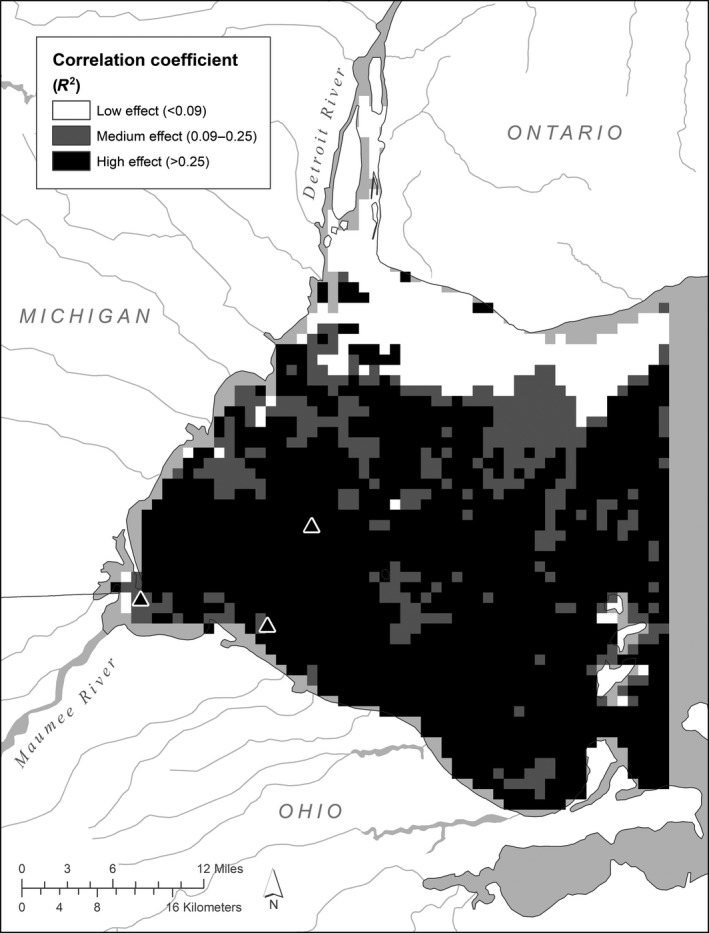
Correlation coefficient (*R* ^2^) of regression models relating March–July total bioavailable phosphorus load to the Maumee River with the maximum annual cyanobacterial index. Data from 2002 to 2016 was used to parameterize the models. Triangles indicate the Maumee Bay and western basin sites that are highlighted in Figure 4

**FIGURE 3:**
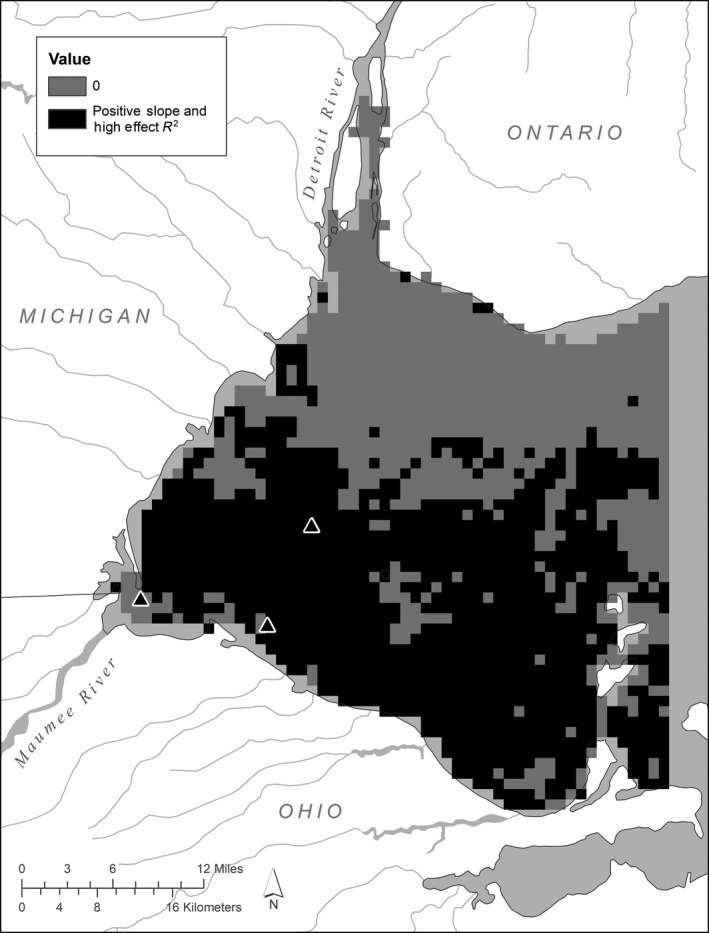
Areas with strong or weak associations between total bioavailable phosphorus load and the cyanobacterial index. Strong associations are inferred by both a high positive slope (>1 *SD* from zero) and a high effect *R* ^2^ value (>.25). Triangles indicate the Maumee Bay and western basin sites that are highlighted in Figure 4

Over the 15 years for which CI data are available, the highest CI values typically occur just north of the mouth of the Maumee River (Figure 1). Many locations near the Detroit River are characterized by an almost complete absence of CI index values above the detection limit (Figure 1). Pixel‐level regressions of TBP load versus CI had *R* ^2^ values ranging from essentially 0 to .83, with the high *R* ^2^ values occurring throughout a wide area of the western basin (Figure 2). Model fit in the Maumee Bay area tended to be lower (*R* ^2^ < .25), although this is an area where maximum CI is often fairly high. Model fit in the areas adjacent to the Detroit River was low, presumably in part because CI values here are rarely above the detection limit. Pixels with both a high *R* ^2^ (>.25) and a slope that was more than 1 *SD* above zero (*a* > 827 cells per ml per metric ton of TBP) accounted for slightly more than half (~1,286 km^2^) of the surface area in the western basin, suggesting this is the spatial extent of strong associations between Maumee River load influence on the western basin (Figure 3).

**FIGURE 4:**
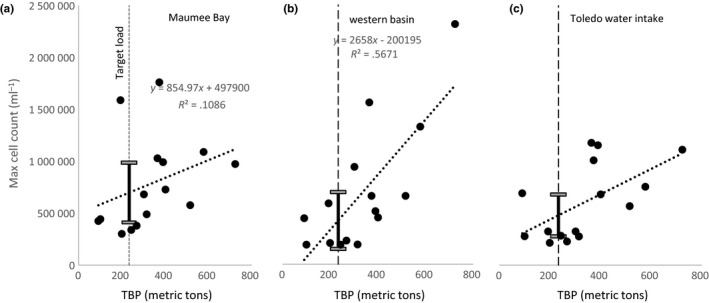
Plots depicting relation between total springtime bioavailable phosphorus load (TBP; metric tons; March 1–July 31) versus the maximum cyanobacterial index (cells per ml) from 2002 to 2016 at three sites in the western basin of Lake Erie. Dotted lines are the simple linear regression. The vertical dashed line is the target load recommended by a group of international experts (Annex 4 Task Team, 2015). Error bars are depicted for the 95% confidence interval of the predicted mean from the simple linear regression at the target load. Sites are (a) Maumee Bay, (b) the open waters of the western basin, and (c) in the immediate vicinity of the Toledo water intake

By focusing on individual points representative of the patterns described above and selected for their contribution to ecosystem services, we can illustrate the difference in the relations and conduct more in‐depth model testing. At a location within Maumee Bay, annual TBP is only weakly associated with maximum cell count (*R* ^2^ = .10, *a* = 855 cells per ml per metric ton of TBP, Figure 4), whereas a location farther out into the western basin demonstrates a strong association between TBP and maximum cell count (*R* ^2^ = .56, *a* = 2,658 cells per ml per metric ton of TBP, Figure 4). At a location near the Toledo water intake, the association between TBP and maximum cell count is still moderately strong, although weaker than farther from shore in the western basin (*R* ^2^ = .34, *a* = 1,222; Figure 4).

For the point within Maumee Bay, models with the potential predictors (nutrients, spring discharge, summer temperature) were not supported over a null model, suggesting other factors are driving maximum CI values in this area (null model ΔAIC_C_ = 0; Table 1). In the area where our spatial analysis identified a strong influence of Maumee River TBP loads farther out into the western basin, we found that the TBP and discharge models have more support (ΔAIC_C_ = 0 and 0.8, respectively) than a null model (ΔAIC_C_ = 9.3) or other potential predictors (Table 1). In the immediate vicinity of the Toledo water intake, models with TBP and discharge (ΔAIC_C_ = 0 and 0.7, respectively) were only slightly more strongly supported than a null model (ΔAIC_C_ = 3) and were more strongly supported than models with nitrogen (Table 1). The effect size (*R* ^2^ and slope) for TBP in the site near the Toledo water intake was lower than in the western basin site (Table 1). While TBP was the strongest predictor at these latter two sites, there was no support for differences between the TBP and discharge model.

**TABLE 1:** Model selection at three sites in the western basin of Lake Erie

Location	Model	ΔAIC_C_	*R* ^2^
Maumee Bay	Null	0	—
	Discharge	0.6	—
	TBP	1.5	—
	TKN	1.8	—
	NO23	2.2	—
	TKN:NO23	3.0	—
	Temperature	3.2	—
Western basin	TBP (*β *= 0.75)	0	.57
	Discharge (*β* = 0.74)	0.8	.59
	TKN	6.8	—
	NO23	7.3	—
	Null	9.3	—
	Temperature	12.3	—
	TKN:NO23	12.5	—
Toledo water intake	TBP (*β *= 0.58)	0	.34
	Discharge (*β *= 0.55)	0.7	.31
	Null	3.0	—
	Temperature	6.1	—
	TKN	15.9	—
	NO23	16.4	—
	TKN:NO23	21.6	—

Note

The response variable in these models is the cyanobacterial index. Models are ordered by their support as inferred from ΔAIC_C_. Discharge refers to average daily discharge (in m^3^) from March 1 through July 31 from the Maumee River. Nutrient loads are all from the Maumee River from March 1 through July 31. NO23, oxidized nitrogen load (metric tons); TKN, total Kjeldahl nitrogen (reduced N); TBP, total bioavailable phosphorus load (metric tons); TKN:NO23, the ratio of the reduced to oxidized nitrogen. Temperature is the average air temperature from March 1 through July 31. Standardized slopes (*β*) and *R* ^2^ values are reported for models that are >2 AIC_C_ units lower than the null model and within 2 AIC_C_ units of the best model.

## 4 DISCUSSION

Our spatial analysis of TBP effects on cyanobacterial abundance in the western basin of Lake Erie found that the localized effects of Maumee River P loading vary widely across the basin with areas of (1) high cyanobacterial abundance but low sensitivity to changing Maumee River P loads (consistent blooms), (2) highly variable cyanobacteria abundance and high sensitivity to changing P loads (strong TBP associations), and (3) minimal cyanobacteria abundance and low sensitivity to changing Maumee River P loads (weak or no TBP associations). These categories appear to reflect the year‐to‐year variance in Maumee River waters. Areas in category 3 appear to be simply outside the area where Maumee River waters tend to mix due to the predominant water movement patterns. These areas tend to be in the northeastern portions of the western basin, which is consistent with results from other studies (Fang et al., 2019; Manning et al., 2019). By contrast, areas in category 1 experience Maumee River waters annually. This means that even in years with low TBP load, the P availability is probably high in these locations, and we might expect legacy P to have accumulated here. The variations that occur annually in TBP load only seem to matter for areas in category 2 (slightly more than half of the basin), which are areas where Maumee River and Detroit River waters mix and Maumee River waters may only extend to these areas during years with high TBP load (and associated high discharge). The net result of these local patterns, at the scale of the entire western basin, is that bloom size and total cyanobacteria abundance vary with TBP. This relation has been previously observed by multiple studies at the whole Lake Erie scale (Bertani et al., 2016; Stumpf, Johnson, Wynne, & Baker, 2016). However, the variety of local relations that we find underlying this whole lake pattern reveals that local observations may not reflect the larger pattern.

Our analysis was not able to distinguish between the relation of CI to Maumee River TBP loading and discharge. Over the 15 years of the study, TBP and discharge are extremely tightly correlated (Pearson's *r* = .97), implying spring concentrations of TBP were relatively consistent over this time period. This strong correlation makes it impossible on a statistical level to separate the effects of TBP and discharge itself. High discharge does not merely bring more TBP to the system, but also influences how large the Maumee River plume is and where it mixes in the western basin (Ludson, Pangle, & Tyson, 2010), which influences where blooms are likely to occur. Current management efforts are focused on reducing the long‐term TBP load, but presumably the discharge will remain the same (or possibly increase; Annex 4 Task Team, 2015; Stow, Cha, Johnson, Confesor, & Richards, 2015), so load reductions will have to take the form of lower P concentrations in Maumee River inputs. Such concentration changes are not captured in the statistical associations explored in our analysis.

This analysis suggests that there are large differences in the relation between P load and cyanobacterial abundance across the western basin of Lake Erie that may influence public experience of the effects of ongoing nutrient management. However, it also suggests several future research directions that are important to fully exploring this pattern and predicting local cyanobacteria abundance. First, as mentioned above, separating the effects of TBP loading and discharge is impossible on a statistical level given the high correlation of these variables in the available Maumee River data. Returning to this analysis as management shifts the P concentration in the Maumee River, and conducting mechanistic scenario modeling to explore this relation will be important to updating our predictions of P loading effects. Second, multiple riverine and point sources contribute to TBP loading to the western basin. We have focused our analysis on the Maumee River as the largest source of high concentration TBP to the western basin, as have other researchers before us (Bertani et al., 2016; Newell et al., 2019). However, especially for the exploration of local effects in nearshore areas, measurements of other TBP sources (e.g., other tributaries, internal loading, wind events, etc.) might be driving annual variation in cyanobacterial abundance. Third, we have focused on spatial differences in patterns of annual maximum CI, and a similar analysis could examine the temporal variability in HABs in the western basin. Length of the HAB season and time specific intensity of the bloom (overlap with swimming or fishing seasons), in addition to maximum bloom biomass, can also affect people's perceptions of the bloom and disruptions of ecosystem services. Do the patterns observed for maximum bloom biomass within the basin or at specific locations also hold across time scales? Finally, the potential uneven distribution of benefits from decreasing TBP loading has implications for human perception of management effectiveness. We speculate on these implications below; however, integrating our findings into a full human societal perspective requires additional research across the disciplines of ecosystem services, economics, sociology, and others.

The local differences in cyanobacteria response to Maumee River TBP loading described here have several potential management implications. Identifying management options for the mitigation or minimization of cyanobacterial blooms in Lake Erie have focused heavily on using the maximum or average cyanobacterial biomass at the whole lake scale as the key response variable indicating the severity of bloom conditions. This focus has led to the conclusion that P load reductions are needed to reduce cyanobacterial effects to Lake Erie (Annex 4 Task Team, 2015; Bertani et al., 2016; Obenour et al., 2014; Stumpf et al., 2012). The current management consensus for reducing the effects of cyanobacterial blooms in Lake Erie is focused on reducing P to a level that would “produce a mild bloom…the size of that observed in 2004 or 2012, or smaller, 90% of the time” (Annex 4 Task Team, 2015), which an international team of experts recommended could be achieved with a reduction in the 2008 spring TBP load of about 40%. In terms of TBP, the 2008 spring load was 389.5 metric tons of P, so a 40% reduction would be 233.7 metric tons of TBP. Over the 15‐year period examined in this study (2002–2016), spring TBP loads fell below that target load value four times (2005–2006, 2012 and 2016). Therefore, a successful 40% reduction in TBP would fall well within the data used in this analysis to construct pixel‐specific regression models.

The implicit assumption in the current management strategy is that reducing the cyanobacterial biomass would reduce the effects of cyanobacteria on ecosystem services to people in the Lake Erie basin. The spatial analysis performed in this paper suggests that reductions in cyanobacterial biomass would occur across slightly more than half of the western basin but mainly in offshore areas. Reductions in cyanobacterial biomass in those areas will no doubt improve conditions for a variety of ecosystem services (Allan et al., 2017). On the other hand, there are many important ecosystem services likely affected by these cyanobacterial blooms that occur in areas where we observed only weak associations with TBP (Allan et al., 2017). Thus, some ecosystem services affected by cyanobacteria will likely see minimal improvement from decreasing spring TBP load. For example, the most famous effect of cyanobacteria in Lake Erie over the past 15 years was caused by cyanobacterial toxins entering the drinking water intake for the city of Toledo (Bullerjahn et al., 2016). Our analysis suggests we could expect reductions in TBP load to have moderately strong effects here, though less than in the open waters, and so the ecosystem services affected by cyanobacteria here will likely benefit from proposed TBP reductions. In contrast, Maumee Bay and the Maumee River mouth are areas that appear to have very high rates of secondary production (thus supporting fisheries and other higher trophic levels; Larson et al., 2016). Secondary production in Maumee Bay is likely affected by cyanobacteria and cyanotoxins (Larson et al., 2018), and cyanobacterial abundance in Maumee Bay seems unlikely to be influenced by proposed reductions in TBP loads.

## 5 CONCLUSIONS

Nearshore areas and river mouths tend to be the focal points of human interaction with large lakes and marine ecosystems (Braden, Won, et al., 2008; Elliott & Whitfield, 2011; Larson et al., 2013). As a result, conditions in the nearshore and in river mouths are likely to strongly influence the actual and perceived ability of large lakes to provide ecosystem services to surrounding communities. Lake management activities, such as P load reductions, even if they are effective at improving conditions lake‐wide, may be considered unsuccessful if they do not improve the provisioning of important ecosystem services or conditions within parts of the lake most used by or visible to humans. This could be the future case in western Lake Erie, where this analysis agrees with many other studies that indicate substantial reductions in P loading will lead to meaningful declines in peak whole‐lake cyanobacterial biomass, which will certainly improve important aspects of Lake Erie's ability to provide ecosystem services (Annex 4 Task Team, 2015; Bertani et al., 2016; Obenour et al., 2014; Stumpf et al., 2012). However, our preliminary analysis suggests that the current P targets might have less benefits in the ecologically and economically important areas near the Maumee river mouth and the city of Toledo, Ohio. Other studies have also found that there are strong spatial differences in the extent to which P loads influence different areas of Lake Erie (Manning et al., 2019). Both this analysis and the analysis of Manning et al. (2019) are based on statistical associations, but future analyses may benefit from incorporating models on water movement to make predictions about where effects are likely to occur.

## Conflicts of Interest

All authors have no conflict of interest to declare.

## AUTHORS' CONTRIBUTIONS

JHL conceived of the work. All authors contributed to the data analysis, drafting, writing, and editing of the work.

## Data Availability

All data used in this study are available in public repositories (as described in Section 2).
